# Cytosine modifications in the honey bee (*Apis mellifera*) worker genome

**DOI:** 10.3389/fgene.2015.00008

**Published:** 2015-02-06

**Authors:** Erik M. K. Rasmussen, Gro V. Amdam

**Affiliations:** ^1^Department of Chemistry, Biotechnology and Food Science, Faculty of Veterinary Medicine and Biosciences, Norwegian University of Life SciencesAas, Norway; ^2^School of Life Sciences, Arizona State UniversityTempe, AZ, USA

**Keywords:** honey bee, methylation, demethylation, 5-hydroxymethylcytosine, social behavior

## Abstract

Epigenetic changes enable genomes to respond to changes in the environment, such as altered nutrition, activity, or social setting. Epigenetic modifications, thereby, provide a source of phenotypic plasticity in many species. The honey bee (*Apis mellifera*) uses nutritionally sensitive epigenetic control mechanisms in the development of the royal caste (queens) and the workers. The workers are functionally sterile females that can take on a range of distinct physiological and/or behavioral phenotypes in response to environmental changes. Honey bees have a wide repertoire of epigenetic mechanisms which, as in mammals, include cytosine methylation, hydroxymethylated cytosines, together with the enzymatic machinery responsible for these cytosine modifications. Current data suggests that honey bees provide an excellent system for studying the “social repertoire” of the epigenome. In this review, we elucidate what is known so far about the honey bee epigenome and its mechanisms. Our discussion includes what may distinguish honey bees from other model animals, how the epigenome can influence worker behavioral task separation, and how future studies can answer central questions about the role of the epigenome in social behavior.

## INTRODUCTION

Since the first honey bee methylome was sequenced in 2010, our understanding of the functional implications of DNA methylation in the honey bee has begun to unfold (Lyko et al., 2010). 5-methylcytosine (5mC) is believed to be involved in alternative splicing, caste differentiation and worker behavioral task separation (Lyko et al., 2010; Flores et al., 2012; Herb et al., 2012). Recently, several other cytosine modifications were discovered in mammalian genomes (Kriaucionis and Heintz, 2009; He et al., 2011; Ito et al., 2011). These modifications are believed to have separate functions from 5mC as they are distributed differently in the genome, and specific reader proteins for one of these modifications exist (Spruijt et al., 2013). Although studies to investigate cytosine modifications other than 5mC in bees have been performed, little is known about their functions and distributions (Cingolani et al., 2013; Wojciechowski et al., 2014). Here we review cytosine modifications and the enzymatic machinery responsible for their generation in different model organisms.

## HONEY BEES

Nutritional cues lead female honey bee larvae into one of two developmental trajectories. The larvae either develop into a queen or into a worker (Winston, 1991). Queens are larger, highly fecund and long-lived (years), while the smaller workers are functionally sterile and shorter lived (weeks, months). Workers show a flexible physiological and behavioral progression that typically starts with care behavior toward siblings (nursing) and culminates in food collection (foraging) weeks later. Nursing is associated with enhanced somatic maintenance and slower aging than foraging (Münch and Amdam, 2010). Yet, foragers can return to nursing tasks, and this behavioral reversion can put age-associated cognitive decline in reverse as well (Baker et al., 2012).

Honey bees, in other words, display a wide range of phenotypes that include complex social caste development and behavior, behavioral shifts, and plasticity of aging. Epigenetic mechanisms are already found to likely play major roles in queen-worker development as well as in worker behavioral progression and reversion (Kucharski et al., 2008; Spannhoff et al., 2011; Herb et al., 2012). These findings put the honey bee forward as a very interesting study organism to investigate the interplay between the social milieu and the epigenome. The use of the honey bee for complex epigenetic research is, furthermore, not diminished by the mainstream models fruit fly (*Drosophila melanogaster*) and nematode (*Caenorhabditis elegans*), since they do not have the full complement of the mammalian epigenetic machinery (**Table 1**).

**Table 1:** Genomic copies of enzymes implicated in DNA methylation and demethylation and presence of epigenetically modified cytosines in select metazoan groups.

Organism	DNMT1	DNMT3	DNMT2	TET	TDG	5mC in CpG	5hmC	5fC	5caC
Nematode							?	?	?
Fly			•	•	•		?	?	?
Aphid	••	•	•	•		•	?	?	?
Jewel Wasp	•••	•	•	•	•	•	?	?	?
Bee	••	•	•	•	•	•	•	?	?
Mammals	•	•••	•	•••	•	•	•	•	•

*Sources: (Kriaucionis and Heintz, 2009; Law and Jacobsen, 2010; Lyko et al., 2010; Walsh et al., 2010; Ito et al., 2011; Cingolani et al., 2013; Beeler et al., 2014) and assembled genomes available at http://blast.ncbi.nlm.nih.gov*.

## EPIGENETIC MACHINERY

DNA methyltransferases (DNMTs) are enzymes that add a methyl group to the 5^′^ carbon of the DNA base cytosine from the donor *S*-Adenosyl methionine (Law and Jacobsen, 2010). DNMT1 is the “maintenance” DNMT that copies the methylation pattern to the newly synthesized strand during DNA replication. DNMT3 is the *de novo* methyltransferase that can methylate specific loci independently of replication. DNMT2 is primarily an RNA methyltransferase that methylates t-RNA^Asp^ (Goll et al., 2006), however, DNA activity has been shown *in vivo* in the fruit fly (Phalke et al., 2009). The *de novo* and the maintenance DNMTs are found in a range of species including honey bees, mammals, aphids, and jewel wasps (**Table 1**). They are catalytically active in the honey bee (Wang et al., 2006), while fruit fly and nematode only contain a single copy of DNMT2. Nevertheless, 5mC originating from DNA has been reported in the fruit fly in both embryos and adult flies (Lyko et al., 2000), suggesting that DNMT2 has some DNA methylation activity *in vivo*. The impact of 5mC in the fruit fly genome is still debated, however (Phalke et al., 2010; Schaefer and Lyko, 2010).

In mammals, the ten eleven translocation (TET) enzyme is responsible for further oxidizing 5mC to 5-hydroxymethylcytosine (5hmC) that again can be oxidized to 5-formylcytosine (5fC), and ultimately 5-carboxylcytosine (5caC) (Tahiliani et al., 2009; He et al., 2011; Ito et al., 2011). 5fC and 5caC are recognized by the thymine DNA glycosylase (TGD), which is a part of the base excision repair pathway of the mammalian cell (Maiti and Drohat, 2011). The TET enzyme together with TDG are probably central to the mammalian active demethylation pathway (Pastor et al., 2013). Mammalian genomes harbor multiple TET enzyme genes, while bees, fruit flies, aphids, and jewel wasps only have one (**Table 1**). The RNA expression levels of the different mammalian TET enzymes vary greatly between developmental stages and cell types. The honey bee TET catalytic domain is catalytically active *in vitro,* and active transcription of the honey bee TET gene has been shown to vary in different stages of development as well as in different adult tissues (Wojciechowski et al., 2014). Interestingly, some species (including fruit fly) that contain only DNMT2 have well conserved TET orthologs, but their activity and function have not been deciphered (Dunwell et al., 2013).

The honey bee genome encodes several core histone modifying enzymes, which are also part of the epigenetic machinery of the honey bees (The Honeybee Genome Sequencing, 2006). However, the impact of and the mechanisms behind histone modifications are beyond the scope of this review.

## 5-METHYLCYTOSINE

**FIGURE 1:**
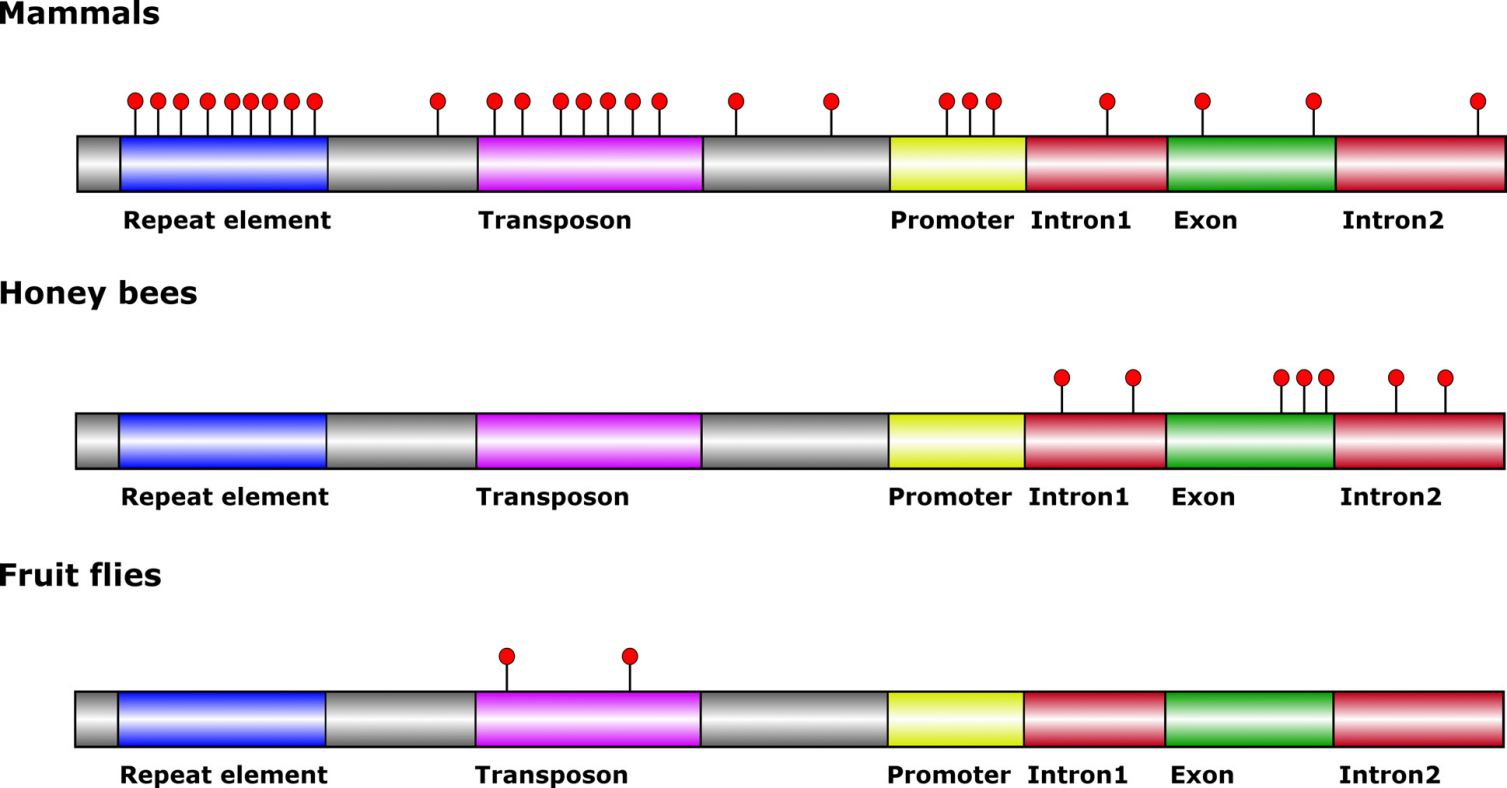
**General features of the 5-methylcytosine distribution in DNA from mammals, honey bees and fruit flies.** Red circles indicate 5-mC. Mammalian genomes are typically methylated in transposon and repeat elements, and at some promoter regions. Intergenic DNA methylation occurs albeit at lower levels. Honey bee genomes are typically methylated in exons close to the exon-intron borders. Non-CpG methylation occurs in introns. Methylation outside of transposons has not been mapped in fruit fly genomes.

The distribution and relative abundance of 5mC vary significantly between mammals, honey bee and fruit fly (**Figure 1**). 5mC is primarily located in a CpG dinucleotide context within repeat sequences and in proximity of promoter areas in mammals (Law and Jacobsen, 2010), whereas in bees methylated CpGs are primarily located within genes (Lyko et al., 2010). However, 5mC can exist in a non-CpG dinucleotide context in both mammals and honey bees (Lister et al., 2009; Cingolani et al., 2013). In addition, the honey bee genome is much more sparsely methylated than mammalian genomes, thus reducing overall complexity and simplifying data analyses for studies conducted in bees. In the fruit fly genome, 5-mC is located within a non-CpG dinucleotide context and seems to be distributed randomly within the genome at an abundance 3- to 100-fold less when compared to honey bees and mammals (Mandrioli and Borsatti, 2006; Phalke et al., 2009). *C. elegans*, on the other hand, does not contain 5mC in its genome (Simpson et al., 1986).

The effect of 5mC on transcription varies between metazoans and genomic context. In mammalian promoters, 5mC is principally a repressive mark, silencing transcription (Bird, 2002). On the other hand, 5mC within gene bodies in mammals, honey bees, and the fruit fly, does not influence transcription levels to the same extent (Mandrioli and Borsatti, 2006; Flores et al., 2012). In honey bees, 5mC within gene bodies rather plays a role in the generation of alternative splice variants on the genome-wide level (Flores et al., 2012; Foret et al., 2012; Li-Byarlay et al., 2013). This role is not clearly defined in mammalian cells, as the role of 5mC in gene bodies differs between cell types and depends on whether 5mC is in a CpG context or not (Lister et al., 2009). These findings make honey bees an attractive system for studies on how 5mC influences the generation of alternative transcripts.

5-methylcytosine is found in multiple cell types, tissues, and life stages in both honey bees and mammals (Ikeda et al., 2011; Ziller et al., 2013). In *D. melanogaster*, 5mC is mostly found during early embryonic stages (Lyko et al., 2000). Although adult 5mC has been reported in fruit fly, the content is too low to be robustly detected by bisulfite sequencing, the gold standard in base resolution 5mC interrogation techniques, making further studies difficult with many established methods depending on bisulfite conversion (Capuano et al., 2014).

## 5-HYDROXYMETHYLCYTOSINE

The TET oxidative products of 5mC recently became a center of attention in mammalian epigenetic research. Many questions about TET and 5hmC dynamics have been answered in embryonic stem cells (Pastor et al., 2013), although 5hmC has been detected in different tissues at different life stages (Kriaucionis and Heintz, 2009; Ivanov et al., 2013). The abundance of 5hmC compared to 5mC is much lower ranging from 2- to 100-fold times less depending on tissue (Kriaucionis and Heintz, 2009; Song et al., 2012). The distribution of 5hmC does not seem to be directly linked to 5mC, as 5hmC is found more often in promoter areas and enhancers, and much less in repetitive elements (Pastor et al., 2011; Stroud et al., 2011; Yu et al., 2012). In addition, proteins capable of specifically binding 5hmC have been discovered, fueling the theory that 5hmC exists as separate epigenetic mark and not simply just as an intermediate in an active demethylation pathway (Frauer et al., 2011; Méllen et al., 2012; Spruijt et al., 2013). In honey bees, 5hmC has been characterized in multiple tissues, and its abundance seems to be highest in germ cells and the brain (7–10% of 5mC and about 4% of 5mC, respectively), following the trend in mammalian cell types (Wojciechowski et al., 2014). Only one study has attempted to map 5hmC in honey bees at a single nucleotide resolution (Cingolani et al., 2013). This same study, surprisingly, mapped the majority of 5hmC in head tissue to non-CpG intronic sequences. Further studies seems warranted to precisely quantify and map 5hmC in bees, especially in non-brain tissue, which has received less interest so far. To date, 5hmC together with 5fC and 5caC have not been identified in the fruit fly, aphid, jewel wasp, and *C. elegans* genomes. However, since *C. elegans* has no 5mC precursor or TET homolog, the existence of 5hmC, 5fC, and 5caC seems highly unlikely.

## 5-FORMYLCYTOSINE AND 5-CARBOXYLCYTOSINE

The recently identified nucleotides 5fC and 5caC have, so far, not accumulated the same level of information as their precursors 5mC and 5hmC. This situation is in part due to extremely low abundance, especially for 5caC, making robust detection difficult (in mammals 5caC is 10- to 1000-fold less abundant than 5hmC). Moreover, the molecular toolbox for investigating 5fC and 5caC is not as developed as it is for 5hmC (Song and He, 2013). Bisulfite sequencing for example, only discriminates between “methylated” and “unmethylated” cytosines, so that 5mC and 5hmC are identified as “methylated” and 5fC and 5caC as “unmethylated” (Pastor et al., 2013). Such data are therefore difficult to use as guidelines in narrowing down possible locations of 5fC and 5caC.

The extremely low abundance of 5caC suggest that this nucleotide is merely an intermediate step in complete demethylation (Song and He, 2013). Although 5fC is a more prominent epigenetic mark than 5caC, its function is still not fully understood. It is possible that 5fC might regulate transcription through stalling of RNA pol II (Kellinger et al., 2012), but further research is needed to elucidate the role of 5fC and 5caC in both vertebrates and invertebrates. In honey bees, 5fC and 5caC have not been investigated yet, though their precursors and catalytic enzyme have been reported (Lyko et al., 2010; Wojciechowski et al., 2014).

## FUTURE WORK: EPIGENETICS AND WORKER BEHAVIOR

Epigenetic mechanisms have been linked to the queen-worker differentiation of honey bees (Kucharski et al., 2008), as well as to worker behavioral progression and reversion (Herb et al., 2012). Herb et al. (2012) bisulfite sequenced brains of age-matched nurses, foragers, and reverted workers (previous foragers now involved in care behavior). Their data revealed differentially methylated regions (DMRs) between the behavioral groups indicating that DNA methylation can play a role in regulation of social behavior. These DMRs are associated with genes involved in development, nuclear pore formation, and ATP binding. RNA sequencing revealed that these same DMRs were connected to alternative splicing events. It is also very likely that the “behaviorally sensitive” DMRs of honey bees are hydroxymethylated at some point during either transition from nurse to forager, or reversion from forager to nurse. Since the study was conducted in adult brain tissue, which has no neurogenesis (Fahrbach et al., 1995), dilution by replication would be unlikely or would only display minor effects. This situation makes these DMRs excellent candidates for investigating if 5hmC is associated with worker behavioral transitions, and if these hydroxymethylated regions are differentially hydroxymethylated between nurses, foragers, and reverted worker bees. Such a study could be the first to establish a putative link between hydroxymethylation and behavior.

Future studies should also dissect the role of TET in worker transitions from nurse to foragers, and back. Other candidate tissues than brain should include the fat body. This tissue is functionally homologous to liver and white adipose tissue and undergoes major remodeling during honey bee behavioral change (Chan et al., 2011). Functional implications of an RNA interference-mediated TET knockdown should provide insight into TET function. Studies can be conducted in honey bee larvae to investigate if TET knockdowns are capable of both queen and worker development. Similarly, consequences for behavioral plasticity can be studied in adult honey bee workers and perhaps link TET and its products with behavior for the first time.

Finally, a possible link between 5hmC and alternative splicing can be investigated by combining 5hmC sequencing at single nucleotide resolution with RNA sequencing of honey bee tissue samples. 5mC is reportedly implicated in the generation of alternative transcripts in the bee, but using methods not able to distinguish 5mC from 5hmC (Flores et al., 2012; Herb et al., 2012). Therefore, further studies that can map 5hmC alongside RNA sequencing data seems warranted, and could potentially give 5hmC a novel function in gene regulation.

## CONCLUSION

The honey bee offers a system where the interplay between DNA methylation and social behavior can be studied in great detail. Published studies of the honey bee epigenome are dominated by questions surrounding queen and worker development, while the epigenetic dynamics of worker behavioral castes have only more recently gained attention. The readily identifiable social behaviors of worker honey bees make setting up precise, large scale experiments feasible (Münch et al., 2013). Better knowledge about honey bee epigenetics also has a dual purpose; increasing the understanding of epigenetic machineries in general, and gaining specific information about gene regulatory mechanisms in an economically important beneficial insect.

## Conflict of Interest Statement

The authors declare that the research was conducted in the absence of any commercial or financial relationships that could be construed as a potential conflict of interest.
